# Cell Bank Origin of MDCK Parental Cells Shapes Adaptation to Serum-Free Suspension Culture and Canine Adenoviral Vector Production

**DOI:** 10.3390/ijms21176111

**Published:** 2020-08-25

**Authors:** Ana Filipa Rodrigues, Paulo Fernandes, Tanja Laske, Rute Castro, Paula Marques Alves, Yvonne Genzel, Ana Sofia Coroadinha

**Affiliations:** 1 iBET, Instituto de Biologia Experimental e Tecnológica, Apartado 12, 2781-901 Oeiras, Portugal; anafr@ibet.pt (A.F.R.); paulo.fernandes@orchard-tx.com (P.F.); laske@mpi-magdeburg.mpg.de (T.L.); rcastro@ibet.pt (R.C.); marques@ibet.pt (P.M.A.); 2 Instituto de Tecnologia Química e Biológica António Xavier, Universidade Nova de Lisboa, Av. da República, 2780-157 Oeiras, Portugal; 3 Bioprocess Engineering, Max Planck Institute for Dynamics of Complex Technical Systems, Sandtorstr. 1, 39106 Magdeburg, Germany; genzel@mpi-magdeburg.mpg.de

**Keywords:** canine adenoviral vectors, influenza virus, MDCK cells, serum-free suspension culture, cell bank repository, transcriptomics, innate immunity, gene therapy

## Abstract

Phenotypic variation in cultured mammalian cell lines is known to be induced by passaging and culture conditions. Yet, the effect these variations have on the production of viral vectors has been overlooked. In this work we evaluated the impact of using Madin–Darby canine kidney (MDCK) parental cells from American Type Culture Collection (ATCC) or European Collection of Authenticated Cell Cultures (ECACC) cell bank repositories in both adherent and suspension cultures for the production of canine adenoviral vectors type 2 (CAV-2). To further explore the differences between cells, we conducted whole-genome transcriptome analysis. ECACC’s MDCK showed to be a less heterogeneous population, more difficult to adapt to suspension and serum-free culture conditions, but more permissive to CAV-2 replication progression, enabling higher yields. Transcriptome data indicated that this increased permissiveness is due to a general down-regulation of biological networks of innate immunity in ECACC cells, including apoptosis and death receptor signaling, Janus kinase/signal transducers and activators of transcription (JAK/STAT) signaling, toll-like receptors signaling and the canonical pathway of nuclear factor kappa-light-chain-enhancer of activated B cells (NF-κB) signaling. These results show the impact of MDCK source on the outcome of viral-based production processes further elucidating transcriptome signatures underlying enhanced adenoviral replication. Following functional validation, the genes and networks identified herein can be targeted in future engineering approaches aiming at improving the production of CAV-2 gene therapy vectors.

## 1. Introduction

Canine adenoviral vectors type 2 two (exhibit a preferential tropism for transducing neurons and present high levels of retrograde transport. These features make them an excellent tool in neurobiology and neuropathophysiology studies, and a powerful gene transfer vehicle for brain gene therapy [1,2]. In addition to gene therapy, these vectors have been used in vaccinations and in oncolytic virotherapy (reviewed in [3]). CAV-2 were developed in the early 1990s to circumvent the pre-existing immunity that limited gene therapy with human adenovirus-based vectors [4]. Similar to human adenoviral vectors (hAdV), helper-dependent (or gutless) CAV-2 were developed [5], allowing the accommodation of high gene cargos (up to 36 kb), reducing immunogenicity of the transduced cells and increasing overall vector safety [6].

Replicative-deficient CAV-2 can be produced in E1-transcomplementing canine cells. This was firstly achieved with dog kidney (DK) cells [4] and later with Madin–Darby canine kidney (MDCK) cells to facilitate the transition from pre-clinical to clinical compatible production processes. In this scope, we established E1-transcomplementing and Cre-expressing MDCK cells [7] that were expanded to a master cell bank under good manufacturing practices (GMP) and implemented CAV-2 production bioprocesses in scalable stirred culture systems [8] and using serum-free media [9]. In addition to its canine origin, MDCK cells were chosen because they are well established, widely used and validated to produce biopharmaceuticals for human healthcare.

MDCK cells were one of the first continuous cell lines to be recommended by the World Health Organization for the replacement of chicken eggs in the production of seasonal influenza vaccines in the mid-1990s [10]. Since then, their potential was assessed and validated by several academic and industrial laboratories, and consolidated with market approval of Flucelvax^®^ and Optaflu^®^ (Novartis), the first cell-culture derived influenza vaccines [11]. In addition to influenza viruses or CAV-2, MDCK cells are known to support the replication of a wide variety of viruses and viral vectors to high titers [12].

MDCK cells were isolated in 1958 by S.H. Madin and N.B. Darby from normal kidney cells of a cocker spaniel dog and, presumably, became spontaneously immortalized during in vitro culture [13]. Although the establishment of these cells was not published, the procedure was similar to that described for Madin–Darby bovine kidney (MDBK) cells and Madin–Darby ovine kidney (MDOK) cells [14]. MDCK cells were submitted, in 1964, at cell culture passage 49 to the precursor of the American Type Culture Collection (ATCC) becoming Certified Cell Line 34 (CCL-34) [15]. The European Collection of Authenticated Cell Cultures (ECACC) is also a main supplier of MDCK parental cells: ECACC 85011435 and ECACC 84121903, originated from different depositors [16].

MDCK parental cells are known to be a heterogeneous cell population [16] and sub-cloning results in clones with distinct properties, including different capacities to support the replication of viruses as reported for influenza virus [17]. Moreover, in respect to adaptation to serum-free suspension cultures, R. van Wielink and colleagues reported an easy procedure resulting in a single cell suspension [18]. However, we have experienced the adaptation of MDCK parental cells to serum-free suspension cultures to be very challenging and to result in cell clumps of variable sizes [9,19]. Since the original MDCK cells were heterogeneous, successive sub-culturing in different laboratories, including ECACC and ATCC, may have resulted in cells with different characteristics. This aspect is important since, in addition to challenging the adaptation to serum-free suspension cultures, such differences can also affect the yield and quality of viral preparations.

In this work, we evaluated MDCK parental cells from ATCC and ECACC for the production of CAV-2 in serum-containing adherent and in serum-free suspension cultures, and conducted whole-genome transcriptome analysis to shed light on the differences between cells from the two sources. To further complement the study, we evaluated the replication of another virus, influenza A virus (IAV). Our results showed that MDCK cells from independent sources differ in heterogeneity, adaptability to serum-free suspension growth and permissiveness for virus replication. The differences in the transcriptome of ECACC vs. ATCC cells further linked increased virus replication permissiveness to a down-regulation of biological networks governing cellular innate immunity.

## 2. Results

### 2.1. Cell Growth and Virus Production in Adherent MDCK Cultures

**Figure 1:**
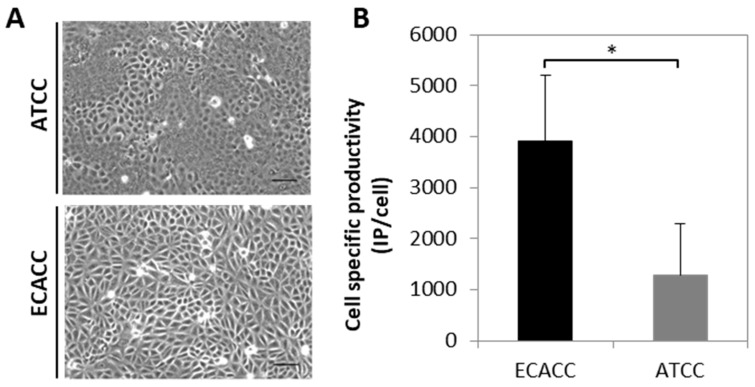
Morphology and canine adenoviral vectors type two (CAV-2) productivity of Madin–Darby canine kidney (MDCK) parental cells from American Type Culture Collection (ATCC) and European Collection of Authenticated Cell Cultures (ECACC). (**A**) Morphology of ATCC (upper panel) and ECACC (bottom panel) MDCK cells in confluent monolayer cultures. Cells were analyzed in the first passages (2 or 3) relative to the original vial received from each cell bank, using an inverted contrast phase microscope and digitally processed using the open source ImageJ software (http://imagej.nih.gov/ij/, 1997–2012). Scale bar: 100 μm. (**B**). Cell specific productivity of CAV-2 with MDCK parental cells from ECACC and ATCC in serum-containing adherent cultures assessed at 40 h post infection. Infection was performed with CAV-2pIX-GFP virus at multiplicity of infection (MOI) 5 and infectious particles titer determined in DK-E1 cells as detailed in Materials and Methods. Values are shown as average ± standard deviation (*n* = 4), * *p* < 0.05, given by Welch’s *t*-test.

An initial comparison of adherent MDCK parental cells from ECACC and ATCC was performed to evaluate cell growth and CAV-2 production. Both cell lines presented similar growth profiles However, to attain ~80% confluence 24 h after seeding, it was necessary to duplicate the inoculum of ATCC cells in comparison to ECACC cells, since ATCC cells tended to grow in clusters. Furthermore, by observing cell monolayers under a contrast phase microscope at full confluence, ATCC cells showed to be morphologically more heterogeneous than ECACC cells (Figure 1A). Both cell lines were also evaluated as substrate for the production of a replicative CAV-2 and those from ECACC showed consistently higher infectious titers (five-fold on average, Figure 1B). The total number of particles, measured in VG/mL, was almost three-fold higher for ATCC cells resulting in CAV-2 preparations with improved ratios of infectious to total particles (IP:TP) when these are produced with ECACC cells.

### 2.2. Adaptation of MDCK Cells to Serum-Free Suspension Growth

MDCK were adapted to two serum-free culture media: Adenovirus Expression Medium AEM and SFM4BHK21. These media were selected because AEM demonstrated to enable appreciable cell growth and CAV-2 production with the MDCK.SUS2 cell line [9] from ECACC, while the SFM4BHK21 medium showed to be suitable to adapt MDCK parental cells from ATCC to grow in single-cell suspension cultures [18].

**Figure 2:**
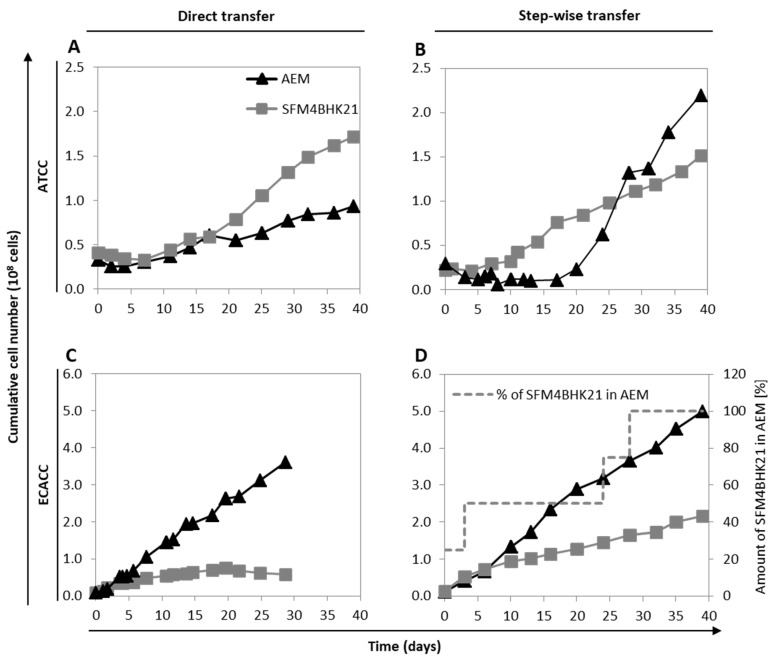
Growth of MDCK parental cells from ATCC or ECACC during adaptation to Adenovirus expression medium (AEM) or SFM4BHK21 media. (**A**,**B**) Cumulative number of ATCC cells following a direct transfer (**A**) or stepwise transfer (**B**) strategy. Stepwise adaptation was performed by first adapting cells to the new serum-free medium in static monolayer cultures and then transfer to suspension cultures. (**C**,**D**) Cumulative number of MDCK.SUS2 cells (from ECACC) following a direct transfer (**C**) or stepwise transfer (**D**) strategy. In the case of MDCK.SUS2, ‘direct transfer’ applies to SFMBHK21 medium only since these cells were already growing in suspension in AEM medium; one AEM culture was kept as control in each adaptation campaign and is shown for comparison purposes. Stepwise adaptation was performed by increasing the percentage of the SFMBHK21 in the final culture medium used to maintain Erlenmeyer cultures. Figure A1 and Figure A2 show the morphology and Figure A3 shows flow cytometry analysis of adapted cells to better characterize macroscopic changes induced by the adaptation (Appendix A, Supplementary Materials). Figure A4 summarizes published MDCK cells derivations (from ATCC or ECACC parental strains) in the context of bioprocess production, including those reported in this work (Appendix A, Supplementary Materials).

To adapt ATCC cells, we started from serum-containing adherent cultures growing in static monolayer, and employed two approaches: (i) direct transfer, where cells growing in static monolayer in serum-containing medium were placed directly into stirred cultures with serum-free culture medium (AEM or SFM4BHK21); or (ii) stepwise transfer, where cells were first adapted to the new serum-free culture medium (AEM or SFM4BHK21) in static cultures prior to being placed into stirred cultures with serum-free culture medium. The results showed that ATCC cells could be directly adapted to grow in suspension with SFM4BHK21 (Figure 2A), while the best adaptation strategy with AEM had to follow the stepwise approach (Figure 2B).

Despite several attempts, MDCK cells from ECACC could not be adapted to grow in suspension in any of these formulations (AEM or SFMBHK21) starting from serum-containing static conditions. Therefore, MDCK.SUS2 cells already growing in suspension as aggregates in the AEM medium [9], were used as ECACC representatives and adapted to grow in suspension in SFM4BHK21 medium. Similar to what was described above, cells were either directly transferred to the SFM4BHK21 medium or stepwise adapted by gradually increasing the percentage of this medium in each subculture passage. When directly transferred to SFM4BHK21 medium, MDCK.SUS2 cells showed no considerable growth during the adaptation phase. Moreover, from day 20 to day 29 there was a slight but continuous reduction in the cumulative cell number indicating cell death (Figure 2C). This contrasted with what was observed with the stepwise approach, in which cells grew continuously from day one of the adaptation phase (Figure 2D). 

### 2.3. Growth of MDCK Cells in Suspension Cultures

**Figure 3:**
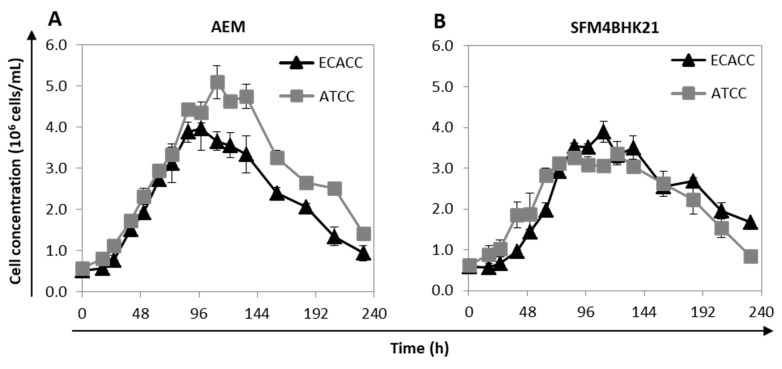
Growth profile of MDCK cells from ATCC or ECACC adapted to AEM or SFM4BHK21 media. Growth profile of selected MDCK cells in suspension cultures with (**A**) AEM and (**B**) SFM4BKH21 media. Cells selected for evaluation were those showing lower adaptation times and a higher cumulative cell number. When using the AEM medium, this corresponded to stepwise transfer for ATCC cells and direct transfer for ECACC cells [9] while in the SFM4BHK21 medium, this corresponded to direct transfer for ATCC cells and stepwise transfer for ECACC cells.

The growth profile of suspension-adapted cells was analyzed in AEM and SFM4BHK21 media. For each medium, the cells selected to be further evaluated were those showing faster adaptation times and higher cumulative cell numbers. When using SFM4BHK21 medium, this corresponded to direct transfer for ATCC cells and stepwise transfer for ECACC cells. With the AEM medium, it corresponded to stepwise transfer for ATCC cells and to what is designated ‘direct transfer’ for the ECACC cells, which is not an actual transfer since we started from MDCK.SUS2 cells already growing in this formulation. ECACC and ATCC cell lines showed similar growth profiles for the same culture medium although, although ATCC cells achieved higher values of maximum cell concentrations in the AEM medium (Figure 3). Overall, AEM was the culture medium in which both MDCK cell lines presented better growth performances.

### 2.4. CAV-2 Prodution and Infection Progression in Suspension Cultures with AEM Medium

**Figure 4:**
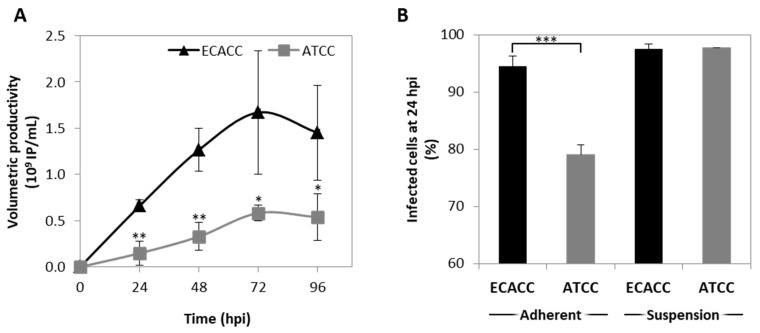
CAV-2 infection permissiveness in MDCK cells from ATCC or ECACC in suspension cultures in AEM medium. (**A**) Volumetric productivity of CAV-2 infectious particles (IP) is given as average ± standard deviation (*n* = 3). Infection was performed with CAV-2pIX-GFP virus at MOI of 1 and infectious particles titer determined in dog kidney (DK)-E1 cells as detailed in Materials and Methods * *p* < 0.05, ** *p* < 0.01, given by a Welch’s t-test, comparing values obtained at the same time point. (**B**) Percentage of GFP^+^ cells at 24 h post infection (hpi) upon transduction with a non-replicative E1-deleted CAV-2 at MOI of 10 for both adherent and suspension cultures to achieve synchronous infection. GFP^+^ cells were considered infected and quantified by flow cytometry. Values are shown as average ± standard deviation (*n* = 3). *** *p* < 0.001, given by a Welch’s *t*-test.

CAV-2 production was evaluated in suspension cultures of ECACC and ATCC cells growing in the AEM medium. Cells were infected at a concentration of 0.8–1 × 10^6^ cells/mL, with medium exchange at infection through centrifugation. The results showed that 72 h post infection was the harvesting time with the highest titers for both cell lines (Figure 4A). CAV-2 titers obtained with ECACC cells were approximately three-fold higher than those obtained with ATCC cells, with a cell specific productivity of 1870 (±651) IP/cell and 827 (±55) IP/cell, respectively.

Since reduced productivity performance of ATCC cells had already been observed in serum-containing static adherent cultures (Figure 1B), we hypothesized that these cells were either less prone to virus entry, or more restrictive in allowing infection progression. To evaluate virus entry, transduction efficiency was estimated by quantifying the percentage of GFP-positive cells after infection with CAVGFP vector. This is an E1-deleted non-replicative CAV-2 viral vector harboring a GFP expression cassette as transgene. Therefore, it does not replicate in these cells reporting only on transduction efficiency directly and, thus, on virus entry indirectly. Adherent cells were also included as control. The results showed less GFP-positive cells when infecting ATCC cells in serum-containing static control cultures although, in suspension cultures, the percentage of GFP-positive cells was similar between ATCC and ECACC (Figure 4B).

**Figure 5:**
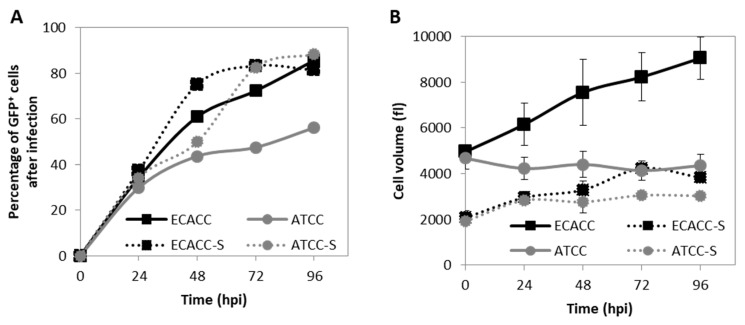
Progression of infection of CAV-2 in MDCK cells from ATCC and ECACC in static monolayer (solid lines) and agitated suspension cultures (S, dotted lines). (**A**) Progression of infection was monitored during the production of CAV-pIX-GFP, a replicative-competent CAV-2, by quantifying the percentage of GFP-positive (GFP^+^) cells by flow cytometric analysis over time. At each time point, parallel non-infected cultures of each the corresponding cells were used as gating controls (see Figure A5, Appendix A, Supplementary Materials for further details). Since CAV-pIX-GFP has a GFP fused to the pIX (capsid protein), GFP expression was used as an indicator of late phase viral protein expression. Values are shown as average (n between 2 and 5) and error bars were omitted for simplicity and visualization clearness. (**B**) Volume of MDCK cells during infection with CAV-pIX-GFP. Adherent cultures were infected at MOI 5 and suspension cultures at MOI 1. Values are shown as average ± standard deviation (*n* = 3).

The progression of CAV-2 infection was evaluated by monitoring the expression of the capsid protein pIX (fused with a GFP), one of the last viral products to be expressed right before the generation of viral particles. Additionally, here, serum-containing adherent cultures were included as control. In static serum-containing adherent cultures, flow cytometry analysis revealed low levels of GFP-positive cells in ATCC cells. CAV-2 infected cells in suspension cultures originating from ATCC achieved higher levels of GFP-positive cells at later time points compared to ECACC (Figure 5A). In addition, ATCC cells showed an atypical CAV-2 replication profile, where the percentage of infected cells seemed to progress more slowly at early time points, relative to ECACC, to then increase abruptly at later time points (Figure 5A). These results also suggest reduced expression levels of the viral proteins, namely pIX, in ATCC cells compared to ECACC’s.

**Figure A3:**
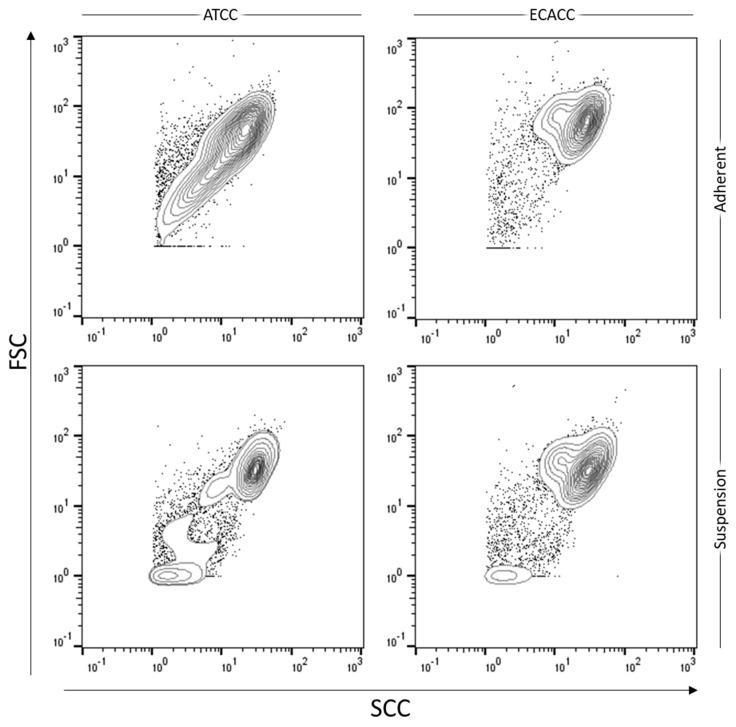
Flow cytometry analysis of MDCK cells from ATCC and ECACC before and after adaptation to serum-free suspension growth. Forward and side scatter (‘FSC vs. SSC’) of ungated cells presented as contour plots showing outliers (dots). Flow cytometry data analysis done in FlowJo^TM^ Software (BD, Ashland, OR, USA).

To gather further evidence on potential restrictions to infection progression in ATCC cells, we analyzed cell size variations since an increase in cell size is a known indicator of infection cycle progression during adenovirus replication [20]. Prior to infection, ATCC and ECACC cells in the same culture system showed similar cell volume (Figure 5B), although cells from suspension cultures were 2.4-fold smaller than those in static cultures. This suggests that adaptation to suspension favored the survival of smaller cells, which is corroborated by flow cytometry analysis (Figure A3, Appendix A, Supplementary Materials). After infection, MDCK cells from ECACC increased in volume in both cultures while no relevant changes were observed for ATCC cells (Figure 5B). These results support that MDCK cells from ATCC and ECACC react differently to CAV-2 infection, with ECACC cells showing more evidence of enhanced infection progression. Together, our analyses corroborate that ECACC cells were less restrictive to infection progression of CAV-2 than ATCC’s, more accentuated in adherent than in suspension cultures.

### 2.5. Transcriptome Analysis

**Figure 6:**
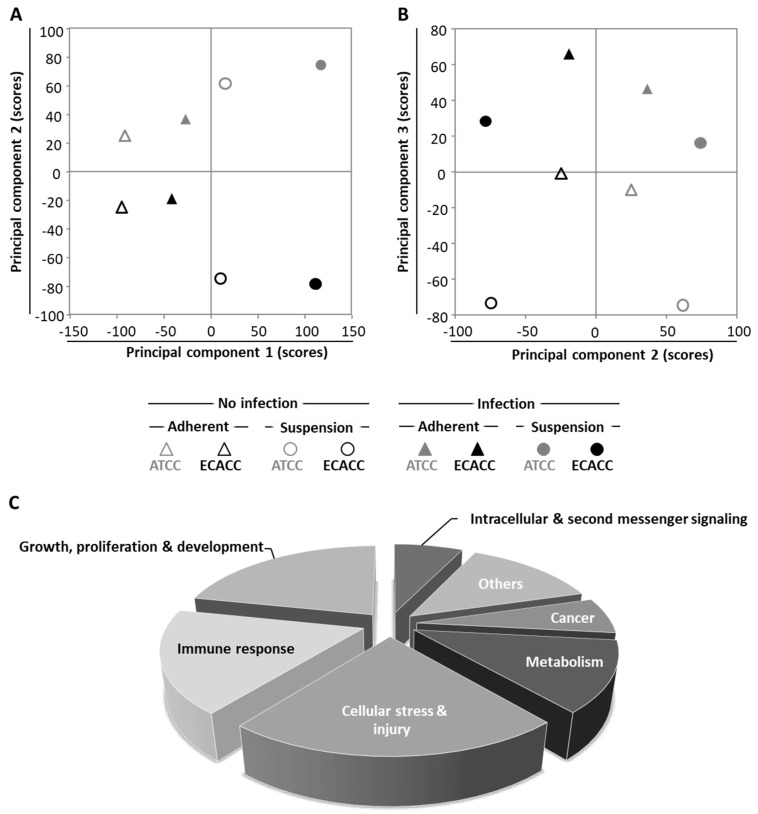
Transcriptome analysis of MDCK cells from ATCC and ECACC under different culture conditions and CAV-2 infection. (**A**,**B**) Scores of principal components (**A**) 1 and 2, and (**B**) and 2 and 3, from a total of 8 principal components identified (Table A1, Appendix A and Supplementary Materials). (**C**) Pathway analysis on ‘infected vs. non-infected cells’ for each pairwise comparison, i.e., matching cell bank origin and culture conditions, using 2-fold as a differential expression cut-off. Significantly enriched pathways were considered for *p* < 0.05 or absolute z-score ≥ 2 in at least one of the pairwise comparisons (Supplementary File 1). Pathway categories adapted from Ingenuity^®^ Canonical Pathways classification. Suspension cultures correspond to cells growing in AEM medium while adherent conditions correspond to cells growing in static monolayer in Dulbecco’s Modified Eagle Medium (DMEM) with 10% of serum (refer to Figure A4, Appendix A, Supplementary Materials, for further details). Infected cells correspond to cells infected with a replicative-competent CAV-2 at 33 h post infection.

To further investigate the differences between MDCK parental cells from ATCC and ECACC, we conducted whole-genome transcriptome analysis. We analyzed infected and non-infected cells from both ATCC and ECACC, cultured either in static and serum-containing medium or in suspension and AEM medium, totaling eight datasets. We started by applying principal component analysis (PCA), an unsupervised data analysis technique that reduces the data dimensionality by creating new variables (principal components, PCs) based on orthogonal transformation that maximizes the variance in the dataset [21]. The first principal components (typically, PC1 to PC5, depending on the dataset size and complexity) capture most of the variance of the dataset, thus providing a good indication of the experimental factors responsible for the differences across samples. Principal component analysis showed that variability is maximized when static serum-containing cells are compared with suspension serum-free cells, separable immediately by PC1, regardless of cell bank origin or infection (Figure 6A): PC1 in the x-axis separates static and serum-containing cultures (on the left side of the axis) from suspension and serum-free cultures (on the right side of the axis). The second layer of variability arises from cell bank origin (Figure 6A, PC2, *y*-axis) followed by CAV-2 infection (Figure 6B, PC3, *y*-axis).

To identify biological pathways relevant in infection, we compared CAV-2 infected with non-infected cells for all matching pairwise comparisons. Differentially expressed genes were analyzed by Ingenuity^®^ Pathway Analysis (IPA). A total of 157 IPA canonical pathways were identified (Supplementary File 1) and assigned to 7 categories (Figure 6C). From these, two categories called our attention: (i) ‘cell stress and injury’ and (ii) ‘immune response’. First because these comprised about 40% of significantly enriched pathways; second because these two categories contain the pathways related to progression of infection, which, according to our previous results and hypothesis (Figure 5) would be a major factor to understand the differences between the two parental cells regarding CAV-2 productivity.

**Figure 7:**
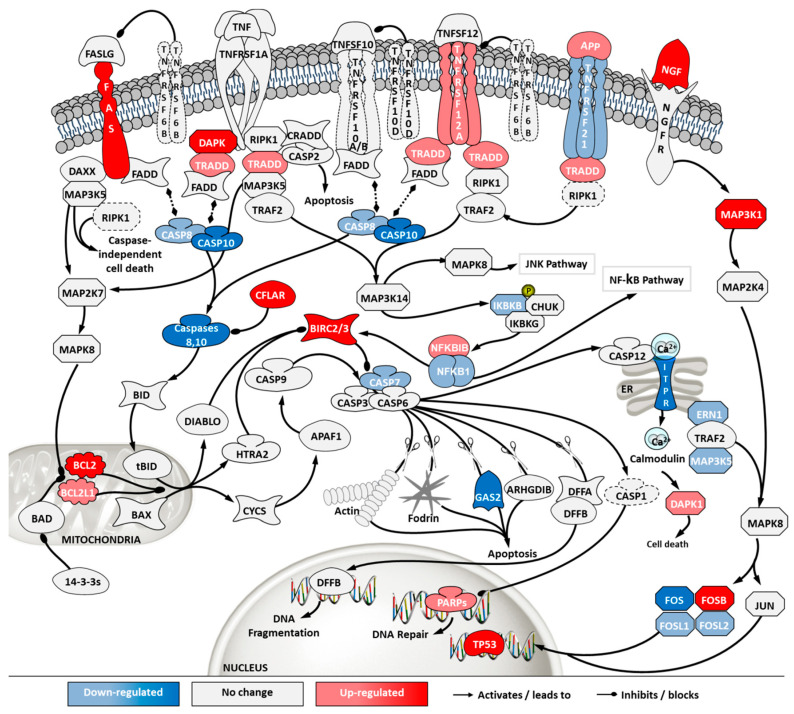
Differential gene expression in apoptosis and death receptor signaling pathway in ‘ECACC vs. ATCC’ cells. Differential expression patterns for pairwise comparison of ‘ECACC vs. ATCC cells’ matching culture conditions and infection status. Differential expression values of 2-fold occurring in, at least one, of the pairwise comparisons are indicated in dark red and dark blue. Since pathway analysis is based on orchestrated changes, even if small, more modest expression changes (between 1.5-fold and 2-fold) are also showing in light red (up-regulation) and light blue (down-regulation), although these modest changes were not used as a differential expression cut-off for running pathway analysis. Dashed genes indicated genes for which no probes were found in the array. Map downloaded from Pathway Central (SABiosciences, now part of Qiagen) and manually curated and adapted. Absolute gene expression values and gene expression fold-change for each gene are given in Supplementary File 3. For a matter of simplicity, only gene symbols are shown: the official full name of each gene was omitted and can be found in Supplementary File 3.

**Figure 8:**
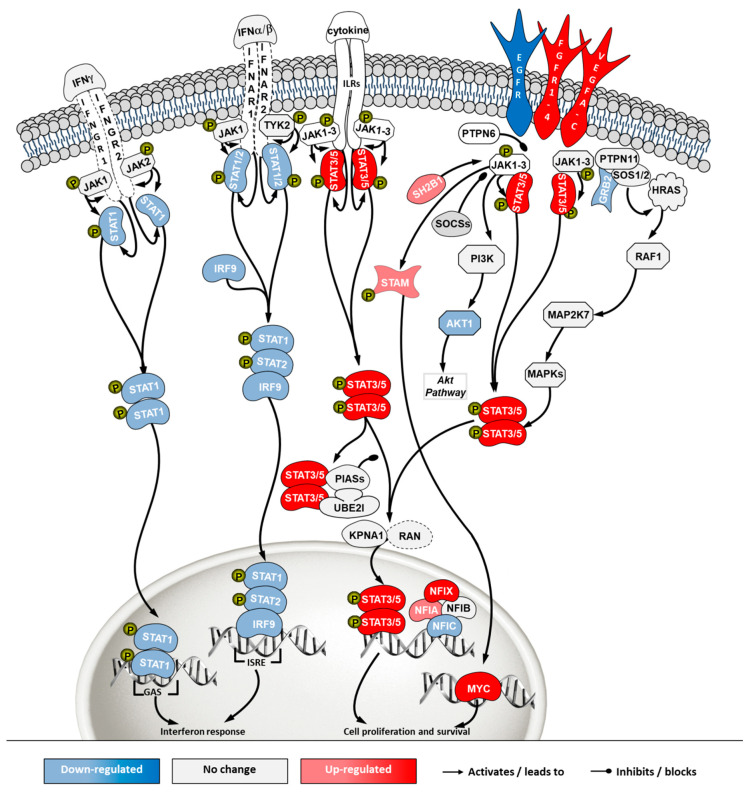
Differential gene expression in Janus kinase/signal transducers and activators of transcription (JAK/STAT) pathway in ‘ECACC vs. ATCC’ cells. For further details refer to the caption of Figure 7.

**Figure 9:**
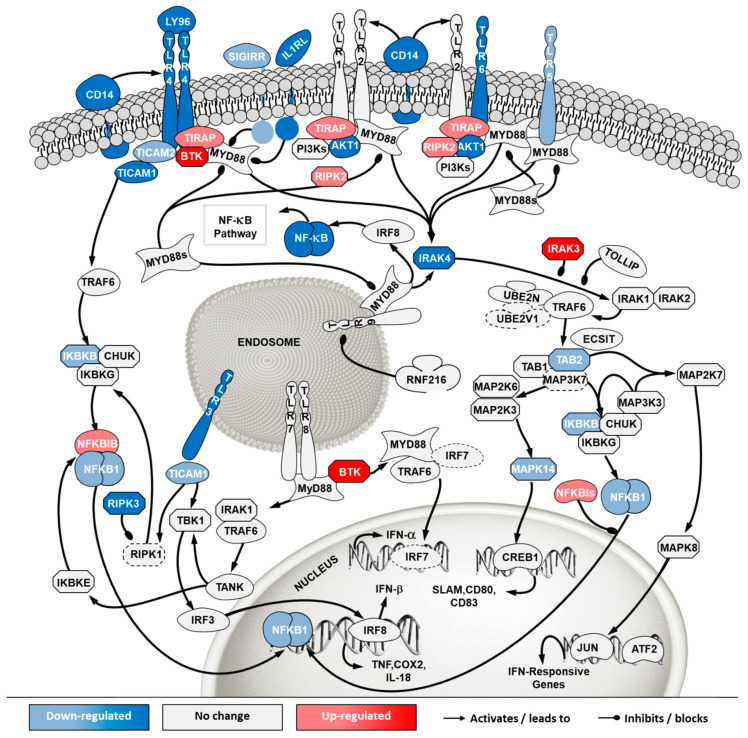
Differential gene expression in Toll like receptor (TLR) pathway in ‘ECACC vs. ATCC’ cells. For further details refer to the caption of Figure 7. NF-κB: nuclear factor kappa-light-chain-enhancer of activated B cells.

**Figure 10:**
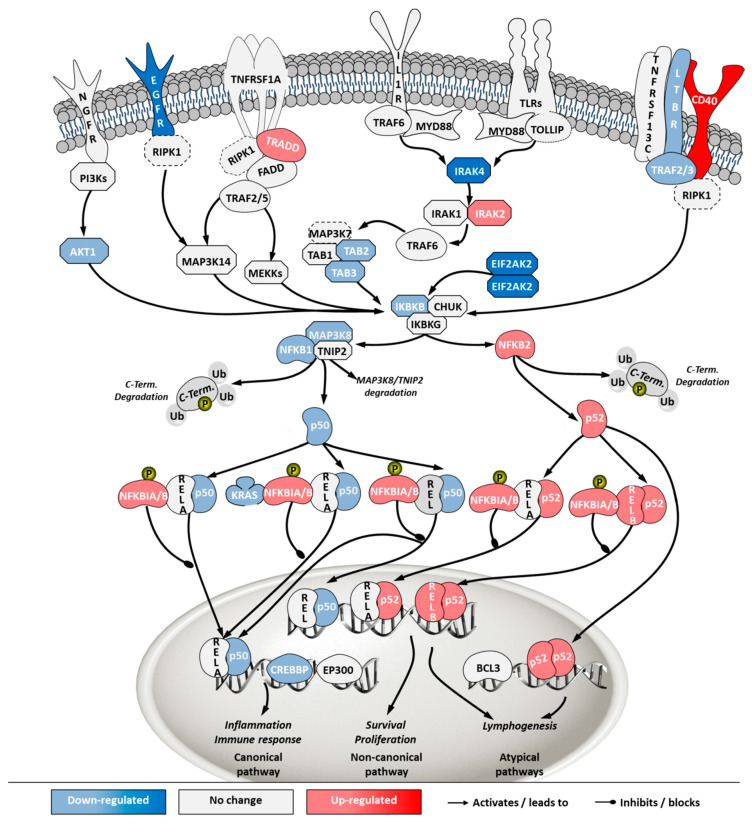
Differential gene expression in nuclear factor kappa-light-chain-enhancer of activated B cells (NF-κB) pathway in ‘ECACC vs. ATCC’ cells. For further details refer to the caption of Figure 7.

To investigate how the pathways in these two categories differ in cells from the two sources, a second pathway analysis was conducted comparing ECACC to ATCC. Among the 296 significantly enriched pathways, 41 corresponded to pathways in the category of ‘cell stress and injury’ or ‘immune response’ with predominance of sub-pathways related to ‘pathogen influenced signaling’, ‘cytokine signaling’ and ‘apoptosis’ (Supplementary File 2). Within these, we further analyzed differential gene expression patterns in the comparison ‘ECACC vs. ATCC’ cells. These patterns are summarized in Supplementary File 3 and were mapped into four pathways (Figure 7, Figure 8, Figure 9 and Figure 10): (i) apoptosis and death receptor signaling; (ii) JAK/STAT signaling; (iii) toll-like receptors signaling and (iv) nuclear factor kappa-light-chain-enhancer of activated B cells (NF-κB) signaling.

Apoptosis and death receptor pathways are involved in programmed cell death in response to external stimuli, including viral infections [22]. Transcriptome data showed that, although ECACC cells had an up-regulation in death receptors (e.g., FAS or TNFRSF12A) and their signaling partners (e.g., TRADD), they presented general down-regulation of apoptotic effectors, namely caspases (Figure 7). In addition, anti-apoptotic proteins such as BIRC, CFLAR or BCL2 and its homologs, were found to be highly up-regulated in ECACC cells relative to ATCC cells, three to five-fold increase (Supplementary File 3).

The JAK/STAT pathway is a central communication system mediating signaling transduction to link response to external stimuli with gene expression. This communication cascade is involved in cellular development, homeostasis and immunity, including a direct link to the interferon signaling pathway [23]. Transcriptome data revealed a general down-regulation of genes in the branch related to interferon response (Figure 8, left side of the pathway) and an up-regulation of the cell survival branch (Figure 8, right side of the pathway) in ECACC cells, relative to ATCC’s.

The toll-like receptor (TLR) signaling is one of the mechanisms of the innate immune system to sense extracellular pathogens [24]. Transcriptome data showed down-regulation of many toll-like receptors in ECACC relative to ATCC cells, most notably TLR4 and TLR6 at the cell surface and known to recognize viral proteins [25]. Important mediators of TLR signaling and its connection to NF-κB canonical pathways were also found to be down-regulated (Figure 9, Supplementary File 3). Additionally, down-regulation of more than three-fold was found for the CD14 antigen, which has also been implicated in the activation of antiviral defense response [26].

Finally, NF-κB pathway was also one of the most highly enriched when comparing ECACC with ATCC cells. The NF-κB pathway is a central transcription regulator of genes involved in immunity, inflammation and cell survival. The pathway is generally subdivided into the canonical pathway and the non-canonical pathway; a third sub-pathway is often considered (alternative/atypical) [27]. The canonical pathway is directly linked to inflammation and the immune response while the others are more connected to cell proliferation and survival. Transcriptome data indicated a down-regulation of genes involved in inflammation and immune response mechanism (canonical pathway) and an up-regulation of genes involved in the cell survival and proliferation mechanisms (non-canonical and atypical) (Figure 10, Supplementary File 3).

Overall, the mapping of differential gene expression in the pathways more enriched in our analysis (Figure 7, Figure 8, Figure 9 and Figure 10, Supplementary File 3) showed expression patterns that point to enhanced resistance to apoptosis and a general down-regulation of innate immunity of ECACC cells relative to ATCC cells.

### 2.6. Influenza Virus Production in MDCK Cells Suspension Cultures with AEM Medium

**Figure 11:**
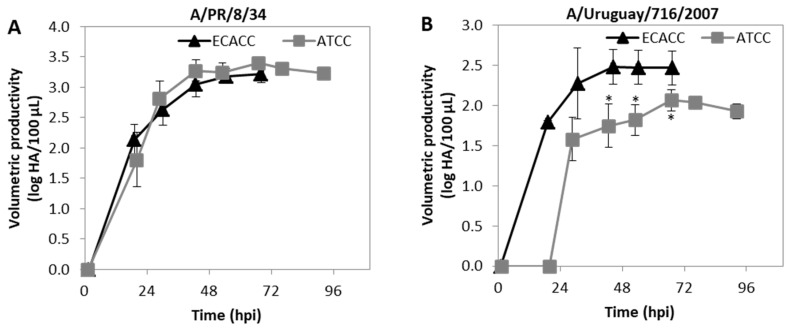
Progression of influenza A virus (IAV) infection in MDCK cells from ATCC or ECACC in suspension cultures of AEM medium. Volumetric titer of total particles (HA titers) over time of (**A**) A/PR/8/34 influenza virus strain and (**B**) A/Uruguay/716/2007 influenza virus strain. Cells were infected at MOI 10^−5^. Values are shown as average ± standard-deviation (*n* = 3). * *p* < 0.05, given by a Welch’s *t*-test comparing values obtained at the same time point.

This work was focused on MDCK cells as hosts for the production of CAV-2. However, MDCK cells are highly relevant in the context of cell-culture based vaccines for influenza virus. We further questioned whether the differences in productivity observed for CAV-2 would occur when cells are used for the production of influenza virus. Therefore, cells were infected at ~2.5 × 10^6^ cells/mL with two influenza virus strains: A/PR/8/34 (RKI) and A/Uruguay/716/2007 (NIBSC). For the A/PR/8/34 (RKI) strain, virus release dynamics assessed by hemagglutination (HA) assay were similar in ECACC and ATCC cells (Figure 11A). Accordingly, both cell lines produced similar maximum infectious titers at the same time (~30 hpi) as assessed by 50 % tissue culture infective dose (TCID_50_) assay (Appendix A, Table A2, Supplementary Materials). The HA and TCID_50_ titers of A/Uruguay/716/2007 (NIBSC) strain produced by ATCC cells showed both a delay in onset of virus release and reduced maximum values (Figure 11B and Appendix A, Table A3, Supplementary Materials). Moreover, the average HA to TCID_50_ ratio for the A/Uruguay/716/2007 (NIBSC) strain produced by either ECACC or ATCC was 13:1 and 112:1, respectively (Appendix A, Table A3, Supplementary Materials) indicating that more non-infectious virions were produced by ATCC cells than by ECACC cells.

## 3. Discussion

Phenotypic variation of cultured cell lines has long been acknowledged and is known to be induced by passage number accumulation, certain culture conditions or even by operator handling [28]. Therefore, the cell bank origin is an expectable source of variability. However, it has been largely overlooked since most researchers conduct their experimental activities using cells from a single source, often, from either ATCC or ECACC cell banks. Previous work from our group to adapt MDCK cells to serum-free suspension growth [9,19] and difficulties to obtain the phenotypes reported by others [18], led us to hypothesize that the cell bank origin was a determinant factor in the final outcome of this adaptation process. Moreover, such differences could be extended to virus production performance, an aspect that was yet to be analyzed. Therefore, in this work, we evaluated MDCK parental cells from ATCC and ECACC for CAV-2 production in adherent and suspension cultures.

A morphological analysis of both cells during the first two to three passages from the original cell bank vials revealed higher heterogeneity of ATCC cells (Figure 1A). This suggests that ATCC cells may be closer to the original population, given that heterogeneity is expected to be reduced with passage number, particularly when fast growing clones overgrow the slower ones. Indeed, although the cells are described as ‘parental’ in both cases, MDCK were deposited first at ATCC and only later at ECACC [16]. Thus, it is expectable that ATCC cells contain subsets functionally similar to ECACC’s. In line with our results, these subsets of cells should be more permissive to virus replication and less likely to survive serum weaning. These functional differences in virus replication have already been demonstrated by sub-cloning ATCC’s MDCK cells, which resulted in clones with different abilities to replicate influenza virus [17]. Heterogeneity differences were also reflected in macroscopic morphological parameters by flow cytometric analysis (Appendix A, Figure A3, Supplementary Materials) although there was no evidence for a link between the morphological and functional differences.

The impact of higher heterogeneity when adapting to serum-free suspension culture is important. In respect to cell physiology, serum removal and anchorage-independent growth is a major change. It mimics an epithelial-to-mesenchymal transition [29,30] which is one of the most complex and multilayer-leveled processes that cells can undergo [31]. In fact, it is not by chance that principal component analysis identified ‘serum-containing adherent vs. serum-free suspension’ cultures as the first source of variability in the dataset, before cell bank repository origin or CAV-2 infection (Figure 6A). During adaptation to serum-free suspension growth, substantial reduction in heterogeneity is expected since only a fraction of the initial cell population will endure the selection process. In this context, starting from higher heterogeneity will always be an advantage to survive in response to external variations and this is probably the reason for which ATCC cells adapted easier and faster. The existence of a plateau during which no variation occurred in the cumulative cell number for at least 10 days, supports that such transition and the corresponding selection process could be taking place in ATCC cells (Figure 2A,B). This ‘selection plateau’ was absent in ECACC cells, which were found to be extremely difficult to adapt and did not grow when directly transferred to new media (Figure 2C). This finding is supported by Bissinger and colleagues, also reporting cell death when directly transferring MDCK.SUS2 (ECACC) to Xeno medium [32]. MDCK parental cells from ECACC seem to require complex culture strategies, such as the already described 10 weeks of serum-weaning plus 10 weeks adaptation in pendulum spinner flasks [19], and longer adaptation processes with stepwise medium variations (Figure 2D). The transcriptome datasets generated in this study, can also be used to better understand the easier adaptation process of ATCC cells to serum-free suspension conditions. However, a direct comparison of ‘ATCC vs. ECACC’ with respect to the adherent-to-suspension transition should be conducted carefully. Because MDCK parental cells from ECACC could neither be adapted to grow in AEM nor in SFM4BHK21 from serum-containing conditions, we had to start from cells already growing in suspension in another serum-free formulation (MDCK.SUS2 [19]). This introduces an intermediate step that does not exist in the adaptation of ATCC cells and, for this reason, we ruled out exploring such analysis herein. Yet, such analysis is possible and can be guided by the proteomics comparison analysis of MDCK.SUS2 and its parental MDCK cell line reported by Kluge et al. [29].

Apart from the serum-free adaptation process, the main purpose of this study was to evaluate the impact of the cell bank origin of MDCK parental cells in CAV-2 production. Differences in productivity between the two parental cells were evident in serum-containing adherent cultures (Figure 1B) and remained in serum-free suspension adapted cells (Figure 4A). These differences seemed to occur with influenza A virus (IAV) production as well, although to a lesser extent (Figure 11). The studies with influenza virus were conducted not only because of the relevance of MDCK in cell-based influenza vaccine production [33,34] but also to evaluate whether the differences in virus production performance between the two cell sources would be maintained. Nevertheless, IAV and CAV-2 are substantially different viruses and direct comparisons should be avoided. This is particularly relevant in the case of transcriptome data of CAV-2 infected cells that does not allow predicting changes in the transcriptome of IAV-infected cells.

The studies on monitoring CAV-2 infection progression (Figure 5) pointed to increased restrictions in viral cycle infection progression in ATCC cells. Further understanding the determinants underlying such differences required a systems-level approach since the production of viruses and viral vectors in mammalian cells is a complex process involving many biological networks [35]. Hence, we used whole-genome transcriptome data and took a pathway analysis approach to identify biological pathways over-represented in the context of virus production. Within these pathways, the main differences between the two parental cells were investigated.

The first pathway analysis, comparing infected with non-infected cells, revealed a predominance of signaling pathways (Figure 6C). This is a characteristic transcriptome signature of adenovirus infection [36], matching fast cellular resources hijacking typical of acute infections, and contrasts with the outcomes of previous analysis of viruses producing chronic infections, such as retroviruses, where metabolic pathways dominate [37]. Among the identified pathways, those related to ‘cell stress and injury’ and ‘immune response’ were the most noteworthy since these categories were likely to contain the biological players related to viral infection progression. Indeed, sub-pathways herein included ‘pathogen influenced signaling’, ‘cytokine signaling’ and ‘apoptosis’. These sub-pathways were explored in-depth and gene expression patterns were mapped for the most relevant ones (Figure 7, Figure 8, Figure 9 and Figure 10).

The control protein E1A (E1A) of adenoviruses has a strong pro-apoptotic effect and infected cells will inevitably undergo apoptosis. This is also the case for E1A of CAV-2 [36]. Although adenoviruses carry their own anti-apoptotic agents [38], including the control protein E1B which is a BCL2 apoptosis regulator homolog, host cells with naturally increased robustness to apoptosis can achieve higher titers by delaying death and enabling more productive use of intracellular resources for the generation and assembly of virions. Therefore, gene expression patterns associated with increased resistance to apoptosis in ECACC cells (Figure 7) are likely related to the higher productivities of these cells relative to ATCC’s. Increased cell survival is in line with the up-regulation of the JAK/STAT pathway branch related to cell proliferation (Figure 8) and the up-regulation of the NF-κB non-canonical pathway (Figure 10). Whether the patterns of increased resistance to apoptosis synergize with or are a consequence of the up-regulation in these other two pathways is unclear.

In addition to increased resistance to apoptosis, the most relevant pathways for the differences in virus production between ECACC and ATCC cells seemed to be those relating to innate immunity systems. Gene expression patterns in these pathways pointed to a reduction in mechanisms of sensing and, most importantly, fighting viral infections. These included a down-regulation of genes in: (i) the JAK/STAT pathway branch related to interferon response (Figure 8), (ii) those involved in toll-like receptors signaling (Figure 9), and (iii) genes in the NF-κB inflammation and immune response canonical pathway (Figure 10). All of these innate immunity-related systems (interferon response, toll-like receptors and NF-κB canonical pathway) provide key mechanisms in fighting adenoviral infections [39]. Interestingly, we found no obvious differences in other well-known innate immunity systems activated by adenoviral infection such as DNA cytosolic sensing mechanisms [39]. This may be related to an insufficient interferon-induced antiviral state reported in MDCK cells since most of these mechanisms depend, at least in part, on interferon signaling [40]. The evident down-regulation of these networks in ECACC cells, relative to ATCC’s, is most likely responsible for the differences in CAV-2 productivity. Moreover, these networks and the identified genes are important target candidates, which, following functional validation, can be used for future genetic engineering approaches to improve the production of CAV-2 for gene therapy. This can be particularly useful in the production of helper-dependent vectors where bioprocess titers are still low.

## 4. Materials and Methods

### 4.1. Cell Lines and Culture Media

Adherent MDCK cells from ECACC (Salisbury, UK) (#84121903) and ATCC (Manassas, VA, USA) (CCL-34) were grown in T-flasks with DMEM (Gibco, Thermo Fisher Scientific, Inc., Waltham, MA, USA) supplemented with 10% (*v*/*v*) fetal bovine serum (FBS) (Gibco, Thermo Fisher Scientific, Inc., Waltham, MA, USA). Another adherent cell line of canine origin E1 transcomplementing dog kidney cell (DK-E1-Cre) was used for CAV-2 seed production and titration of infectious particles as described in [4]. Suspension cells were grown in shaker flasks at 130–150 RPM with AEM (Gibco, Thermo Fisher Scientific, Inc., Waltham, MA, USA) or SFM4BHK21 (Hyclone, Cytiva, Marlborough, MA, USA) supplemented with 4 mM glutamine (Gibco, Thermo Fisher Scientific, Inc., Waltham, MA, USA). Cells were maintained at 37 °C and 5% or 8% CO_2_, for adherent and suspension cultures, respectively, and split twice a week.

### 4.2. Viruses

CAV-2pIX-GFP and CAVGFP are derived from the CAV-2 strain Toronto A 26/61, GenBank J04368. CAV-2pIX-GFP is a replication competent virus with a C-terminal fusion of eGFP in protein IX [41] and CAVGFP [4] is an E1-deleted (ΔE1) vector containing an eGFP expression cassette. Viral vectors stocks were prepared and purified by CsCl gradients as described previously [4,7].

Influenza virus strains used in this study were the H1N1 A/Puerto Rico/8/34 (A/PR/8/34), a wild type strain obtained by the Robert Koch Institute (Berlin, Germany) and the high-growth reassortant NYMC X-175c with an A/PR/8/34-backbone (H3N2 A/Uruguay/716/2007-like ).

### 4.3. Adaptation to Serum-Free Media and Suspension and Growth Assays

Cells were adapted to grow in suspension with serum-free medium using a direct or a stepwise approach. Direct adaptations were performed by placing cells from adherent cultures in DMEM supplemented with FBS directly in final serum-free medium in shaker flasks. Cells from adherent cultures were pelleted by centrifugation (300× *g*, 10 min, 4 °C) and suspended in serum-free medium at 1 × 10^6^ cells/mL. Culture medium was replaced every 2–3 days until cell growth became evident. Once cell concentration reached 2 × 10^6^ cells/mL, 0.5 × 10^6^ cells/mL were seeded in fresh medium. Cells were considered adapted when a maximum cell concentration of 2 × 10^6^ cells/mL was attained in 3 days without medium exchange in at least 3 subsequent passages.

Stepwise adaptations were performed by first adapting cells to serum-free medium (SFM) in adherent cultures and then transfer these SFM-adapted cells to shaker flasks. Adaptation in adherent cultures was performed by gradually increasing the ratio of serum-free-to-serum-containing medium during 3 to 6 subsequent passages. SFM percentage in culture medium was increased when cells in adaptation presented similar growth of adherent cells in DMEM kept in parallel cultures. When cell growth became slower, percentage of SFM was maintained in two consecutive passages. Cells were considered adapted when 90% confluence was reached within 3–4 days in 3 consecutive passages in 100% SFM. Suspension cultures of SFM-adapted cells were maintained as mentioned above.

Growth of suspension cells was assayed using an inoculum of 0.5 × 10^6^ cells/mL in 500 mL shaker flasks with a working volume of 150 mL. Growth was monitored for 10 days by determining cell concentration and viability. All parameters were determined at least in 24 h intervals.

### 4.4. Cell Permissiveness to CAV-2 Infection

To estimate the infection efficiency of CAV-2 in the different cells, cells were infected with CAVGFP using a multiplicity of infection (MOI) of 10 IP/cells with medium exchange at the time of infection. GFP-positive cells were determined at 24 h post infection through flow cytometric analysis (CyFlow Space, Partec, Münster, Germany). The infection assays in adherent cultures were performed in 6-well plates. For these, MDCK cells from ECACC and ATCC were seeded at 1.5 × 10^4^ cells/cm^2^ and 3 × 10^4^ cells/cm^2^, respectively, to be infected the day after with a confluence of ~80% in a working volume of 2 mL/well. Infection assays in suspension cultures were performed in 125 mL shaker flasks, seeding 5 × 10^5^ cells/mL in 20–25 mL working volume. Cells were infected 24–36 h later, when cell concentration was at 0.8–1 × 10^6^ cells/mL.

### 4.5. Progression of Infection and Production of CAV-2

The production of CAV-2pIX-GFP was used to evaluate virus productivity. The expression of capsid protein IX after CAV-2pIX-GFP infection was used to also evaluate the progression of CAV-2 infection by flow cytometry (CyFlow Space, Partec, Münster, Germany). Adherent and suspension cultures for these assays were prepared as described above. Adherent cultures were infected at MOI 5, while suspension cultures at MOI 1. Sampling and cell monitoring were performed at 24 h intervals. At each time point, parallel non-infected cultures of each of the corresponding cells were used as gating controls. Viruses were collected by disrupting cells with lysis buffer (Tris/HCl 10 mM, pH 8.0 and 0.1% (*v*/*v*) Triton-X 100). The resulting sample was clarified at 3000× *g* for 10 min at 4 °C and stored at −85 °C until further analysis.

### 4.6. Production of Influenza Virus

Suspension MDCK cells cultivated in AEM were grown to a cell concentration of 2.5 × 10^6^ cells/mL in 150 mL vented shake flasks (75 mL working volume). For infection, cells were first pelleted (150× *g*, 5 min) and resuspended in a mixture of 95% (*v*/*v*) fresh and 5% (*v*/*v*) conditioned medium containing the virus seed at MOI 10^−5^ virions/cell. To obtain optimal infection conditions, trypsin concentration had to be adjusted for each cell. For MDCK ATCC and ECACC cells, trypsin was added at 5 × 10^−6^ units/cell and 2.5 × 10^−6^ units/cell, respectively. Shake flasks were sampled twice per day for cell counting. Supernatant was clarified by centrifugation (300× *g*, 10 min) and stored at −85 °C until further analysis.

### 4.7. Cell Concentration and Size

Cells were counted using a Fuchs-Rosenthal hemocytometer chamber and viability determined by the trypan blue exclusion method in the majority of the assays. For influenza virus experiments, trypan blue exclusion was automated using a ViCELL™XR device (Beckman Coulter, Indianapolis, IN, USA). In adherent cultures, cells were counted directly from cell suspension after trypsin detachment. For suspension cell lines growing in aggregates, trypsin was used to obtain single cell suspensions. Briefly, from 1 mL sample, cells were centrifuged for 10 min at 300× *g* and the supernatant removed (900 μL). Then, same volume of trypsin was added, cells were incubated at 37 °C until evident detachment, suspended by pipetting up and down and finally counted. Suspension cultures with single cells were counted directly from the sample taken from shaker flasks.

Cell diameter and volume was determined using a CASY^®^ Cell Counter (Schärfe Systems, Reutlingen, Germany). The appropriate program was established according to the instructions of the manufacturer. For measurement, 50–100 µL of cell suspension were transferred to a CASY^®^ cup containing 10 mL CASY^®^ton, mixed by inverting three times and placed in the CASY^®^ Cell Counter.

### 4.8. CAV-2 Quantification

Quantification of infectious CAV-2 particles (IP) was performed by monitoring the expression of GFP by flow cytometric analysis of DK cells subjected to serial dilutions of viral samples, as described previously [7]. Quantification of genome-containing CAV-2 particles (VG) was performed by quantitative real-time PCR, as described previously [42]. Briefly, viral genomes were extracted and purified by High Pure Viral Nucleic Acid Kit (Roche Diagnostics, Penzberg, Germany), and SYBR Green I dye chemistry was used to detect PCR products using LightCycler system. Primers against the GFP gene were used: forward 5′-CAGAAGAACGGCATCAAGGT-3′ and reverse 5′-CTGGGTGCTCAGGTAGTGG-3′.

### 4.9. Influenza Virus Titer Determinations

Determination of total influenza virus concentration was performed with a hemagglutination assay as described before [43]. Read-out is log_10_ HAU/100 µL with a relative standard deviation of the method of 9.3% and a maximum error of ±0.2 log_10_ HAU/100 µL. This can be converted into virions/mL by:

(1) $$\mathrm{Total}\;\mathrm{virus}\;\mathrm{concentration}=2\times 10^{7}\times 10^{\log_{10}\;\mathrm{HAU}/100\;\mu L}$$

Infectious virus particle concentration was determined with a TCID_50_assay as described by Genzel and Reichl [44]. Titer calculations were performed according to the Spearman–Kärber method. The limit of the detection was 3.2 × 10^2^ virions/mL and dilution error was ±0.3 log_10_. For calculations of cell-specific virus yields the maximum virus titers were divided by the maximum cell concentration.

### 4.10. RNA Extraction for Transcriptome Analysis

Total RNA for transcriptome analysis was extracted at 33 h post-infection with a replicative-competent CAV-2. RNeasy Mini Kit (Qiagen, Valencia, CA, USA) for total RNA extractions was used according to the instructions of the manufacturer. Total RNA pellet was eluted in 100 µL of nuclease-free water (Qiagen, Valencia, CA, USA) and stored at −85 °C until further processing. RNA yields were quantified using NanoDrop 2000 (Thermo Scientific, Waltham, MA, USA) and RNA quality was characterized by the quotient of the 28S to 18S ribosomal RNA electropherogram peak using an Agilent 2100 bioanalyzer and the RNA Nano Chip (Agilent, Santa Clara, CA, USA).

### 4.11. Amplification, Labelling and Hybridization of RNA Samples for Microarray Analysis

Whole transcriptome was analyzed by Affymetrix Canine Gene 1.1 ST Array Strip microarray technology (Life Technologies, Carlsbad, CA, USA). To that end, Affymetrix Genechip WT Plus Reagent Kit (Santa Clara, CA, USA) was used to transcribe 100 ng of RNA. After second-strand synthesis, double-stranded cDNA was used in an in vitro transcription (IVT) reaction to generate cRNA (WT Amplification kit Module, Affymetrix, Life Technologies, Carlsbad, CA, USA). A total of 15 µg of this cRNA was used for a second cycle of first-strand cDNA synthesis (WT Amplification kit Module; Affymetrix, Life Technologies, Carlsbad, CA, USA). A total of 5.5 µg of single stranded cDNA was fragmented and end-labelled (GeneChip WT Terminal Labelling Kit, Affymetrix, Life Technologies, Carlsbad, CA, USA). The size distribution of the fragmented and end-labelled cDNA, respectively, was assessed using an Agilent 2100 Bioanalyzer with an RNA 6000 Nano Assay. A total of 3.5 µg of end-labelled, fragmented cDNA was used in a 150-µL hybridization cocktail containing added hybridization controls (GeneAtlas Hybridization, Wash, and Stain Kit for WT Array Strips and GeneChip Expression 3′-Amplification Reagents Hybridization Control Kit, Affymetrix, Life Technologies, Carlsbad, CA, USA) and hybridized on array strips for 20 h at 48 °C. Standard post hybridization wash and double-stain protocols (GeneAtlas Hybridization, Wash, and Stain Kit for WT Array Strips; Affymetrix, Life Technologies, Carlsbad, CA, USA) were used on an Affymetrix GeneAtlas system, followed by scanning of the array strips.

### 4.12. Transcriptome Data Processing and Analysis

Data pre-processing and analysis was done in Affymetrix^®^ Expression Console™ Software using Robust Multi-array Average (RMA)-Sketch at gene level normalization. Differentially expressed genes were considered based on 2-fold expression difference for each comparison analysis considered and evaluated for pathway analysis using Ingenuity Pathway Analysis (IPA) software (Ingenuity^®^ Systems, www.ingenuity.com). Normalized data was also used for principal component analysis using R software [45]. The entire microarray dataset was submitted to ArrayExpress of EMBL-European Bioinformatics Institute with the accession number E-MTAB-9379.

### 4.13. Statistical Analysis

Statistical analysis to compare ATCC and ECACC cells in respect to infectious viral titers was carried out using the Welch’s *t*-test.

## 5. Conclusions

This work shows that MDCK parental cells from ECCAC are a less heterogeneous population, most likely, a sub-population of the parental cells deposited at ATCC. While this sub-population is less prone to adapt to suspension and serum-free culture conditions, it was found to be more permissive to virus replication progression. Transcriptome data indicates that this increase in permissiveness is due to a general down-regulation of biological networks of innate immunity in ECCAC cells, such as apoptosis and death receptor signaling, JAK/STAT signaling, toll-like receptors signaling and NF-κB signaling. Overall, this work shows how different cell origins can shape the outcome of viral-based production processes, further elucidating the transcriptomic profile behind enhanced adenovirus replication.

## Figures and Tables

**Figure A1:**
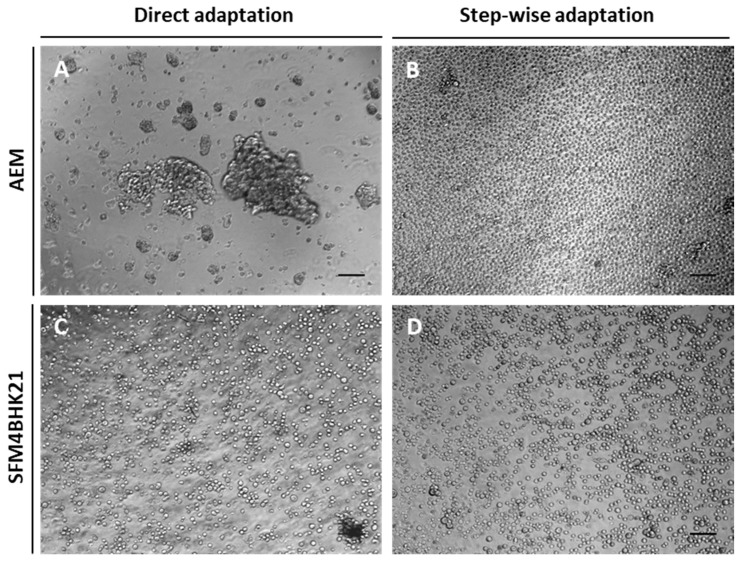
Morphology of MDCK cells from ATCC after adaptation to suspension. Cells were adapted to one of two serum-free culture media—AEM or SFM4BHK21—following a direct approach (**A**,**C**) or a stepwise approach (**B**,**D**). Cells were analyzed and digitally visualized as described in Figure 1. Scale bar—100 μm.

**Figure A2:**
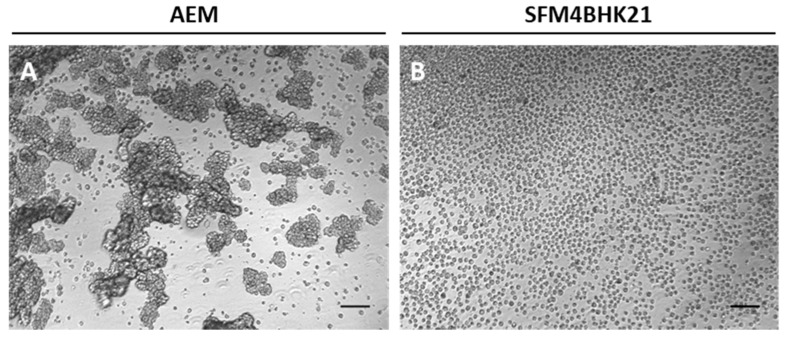
Morphology of MDCK.SUS2 (ECACC) after adaptation to suspension. (**A**) MDCK.SUS2 cells previously adapted to suspension and growing as aggregates in AEM [9], were used as ECACC supplier representative. Direct adaptation of MDCK.SUS2 to SFM4BHK21 medium did not enable cell growth, only the stepwise approach ensured cell survival and resulted in single cell cultures (**B**). Cells were analyzed and digitally visualized as described in Figure 1. Scale bar—100 μm.

**Figure A4:**
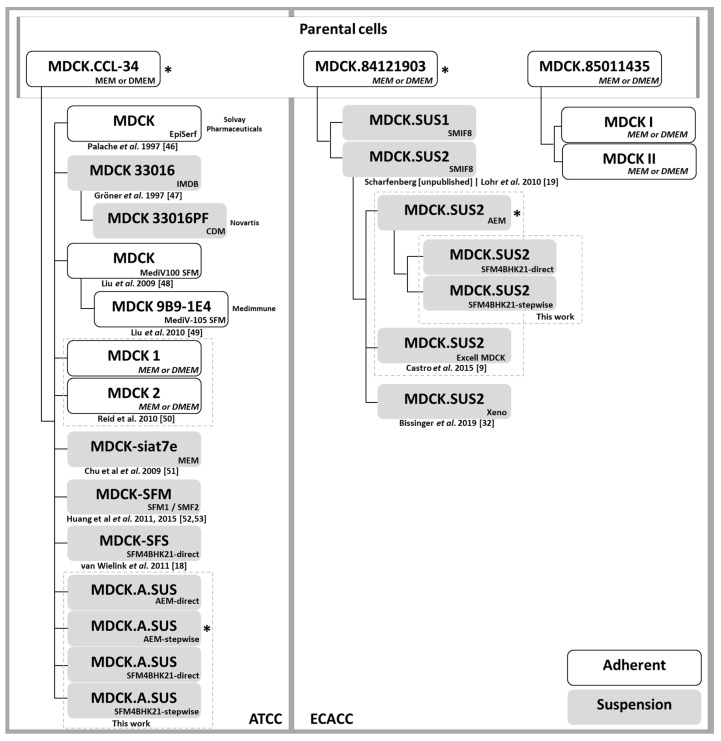
Derivations of MDCK parental cells from ATCC or ECACC cells for cell-cultured based bioprocesses. When that information was available, the use of these cells by pharmaceutical companies (typically for influenza virus production) is indicated. Medium formulation used in each case is indicated in each box on the bottom-right corner. The cells used in this work for transcriptome analysis are indicated with ‘*’ [9,19,32,46,47,48,49,50,51,52,53].

**Figure A5:**
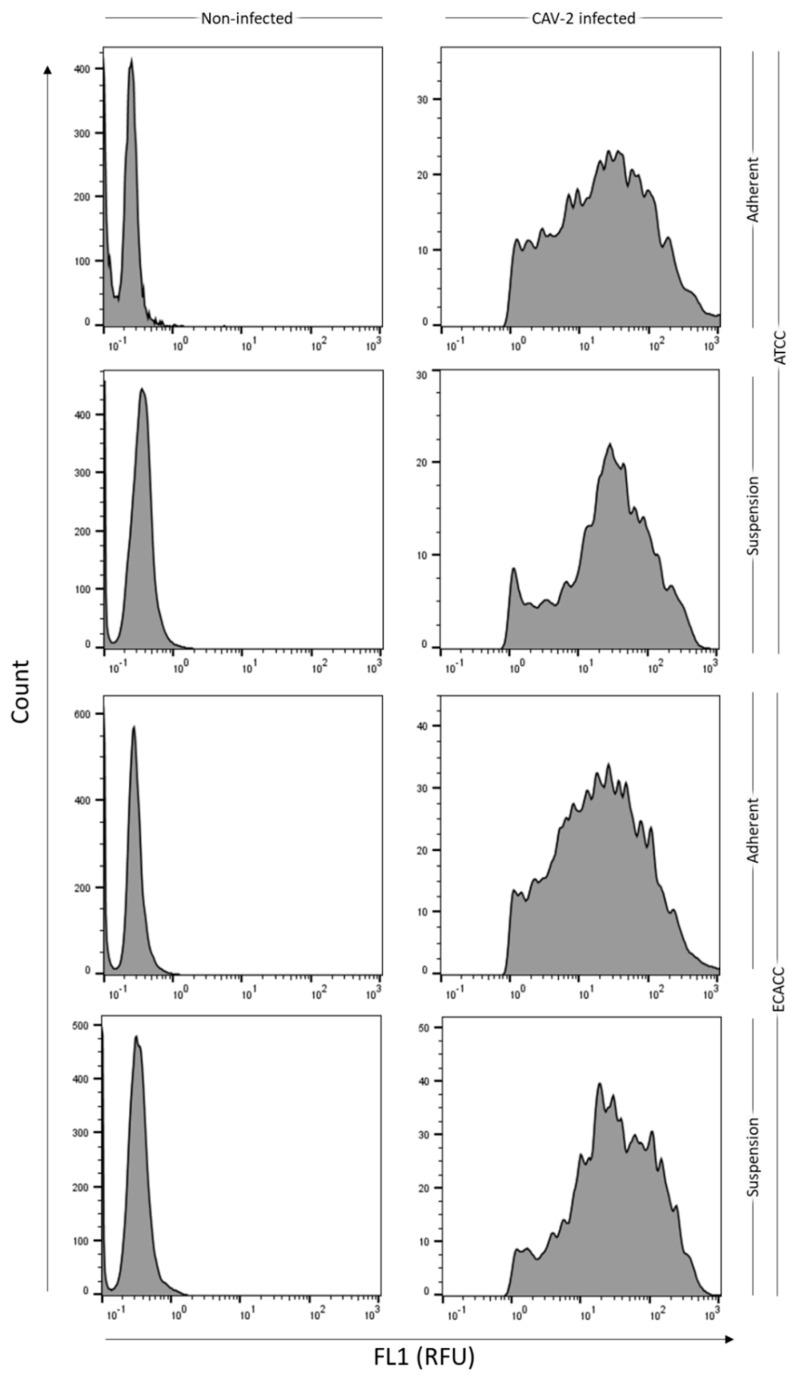
Gating parameters on the monitoring of infection progression by flow cytometry analysis. Non-infected cells (left panels) were used as negative controls for gating and up to 2% false positives in these control samples were considered acceptable. Data corresponds to representative histograms from the 24 h post-infection time-point (refer to Figure 5A from the main manuscript). This time point was chosen as benchmark since fluorescence intensity values are comparable between samples, while later timepoints may be affected, to more or less extent, by superinfection events. Suspension cultures correspond to cells growing in AEM medium while adherent conditions correspond to cells growing in static monolayer in DMEM with 10% FBS. RFU: relative fluorescence units.

## Supplementary Materials

Supplementary materials can be found at https://www.mdpi.com/1422-0067/21/17/6111/s1.

## Abbreviations

AEM	Adenovirus expression medium
ATCC	American Type Culture Collection
BCL2	BCL2 apoptosis regulator
BIRC	Baculoviral IAP repeat containing
CAV	Canine adenoviral vector
CD14	CD14 molecule
CFLAR	CASP8 and FADD like apoptosis regulator
DK	Dog kidney (cells)
E1A	Control protein E1A
E1B	Control protein E1B
ECACC	European Collection of Authenticated Cell Cultures
FAS	Fas cell surface death receptor
GFP	Green fluorescent protein
GMP	Good manufacturing practices
HA	Hemagglutination
hAdV	human adenoviral vectors
HPI	Hours post infection
IP	Infectious particle
IPA	Ingenuity Pathway Analysis
JAK/STAT	Janus kinase/signal transducers and activators of transcription
MDBK	Madin-Darby bovine kidney (cells)
MDCK	Madin-Darby canine kidney (cells)
MDOK	Madin-Darby ovine kidney (cells)
MOI	Multiplicity of infection
NF-κB	Nuclear factor kappa-light-chain-enhancer of activated B cells
NIBSC	National Institute for Biological Standards and Control
PCA	Principal component analysis
RFU	Relative fluorescence units
RKI	Robert Koch-Institut
SFM	Serum-free medium
TCID50	Fifty-percent tissue culture infective dose
TLR	Toll-like receptor
TNFRSF12A	TNF receptor superfamily member 12A
TRADD	TNFRSF1A associated via death domain
VG	Viral genomes

## Appendix A

**Table A1:** Principal component analysis scores.

Culture System ^Δ^	Cell Bank	Infection Status ^ΔΔ^	Scores							
			PC1	PC2	PC3	PC4	PC5	PC6	PC7	PC8
Adherent	ATCC	Non-infected	−91.6	24.9	−10.0	39.0	−51.5	−7.7	−37.8	−3.7 × 10^−14^
Adherent	ATCC	Infected	−26.7	36.4	46.1	65.6	36.9	−6.0	31.7	2.6 × 10^−14^
Adherent	ECACC	Non-infected	−95.0	−24.8	1.4	−48.6	−35.2	3.2	42.1	−7.0 × 10^−15^
Adherent	ECACC	Infected	−41.4	−19.1	65.7	−45.0	46.3	6.6	−33.0	1.8 × 10^−13^
Suspension	ATCC	Non-infected	15.2	61.5	−74.4	−10.8	23.7	49.6	−1.3	−1.4 × 10^−13^
Suspension	ATCC	Infected	117.4	74.3	16.0	−31.4	−21.0	−38.4	1.0	−3.8 × 10^−14^
Suspension	ECACC	Non-infected	10.3	−74.8	−73.2	8.0	30.8	−42.1	−2.3	9.9 × 10^−14^
Suspension	ECACC	Infected	111.8	−78.5	28.4	23.3	−30.0	34.8	−0.5	1.1 × 10^−14^

^Δ^ Suspension cultures correspond to cells growing in AEM medium while adherent conditions correspond to cells growing in DMEM supplemented with 10% FBS. ^ΔΔ^ Infected cells correspond to cells infected with a replicative-competent CAV-2 at 33 h post-infection.

**Table A2:** Maximum tissue culture infective dose (TCID_50)_ titers obtained during the production of influenza virus strains in suspension cultures with MDCK cells derived from ATCC and ECACC.

Strain	Replicate	TCID_50_/mL		FC_ECACC/ATCC_
		ECACC	ATCC	
A/PR/8/34	#1	1.3 × 10^9^	1.8 × 10^9^	0.8
	#2	4.2 × 10^9^	2.4 × 10^9^	1.8
	#3	7.5 × 10^9^	5.6 × 10^9^	1.3
	average	4.4 × 10^9^	3.3 × 10^9^	1.3
A/Uruguay/716/2007	#1	1.0 × 10^9^	5.6 × 10^7^	17.8
	#2	4.2 × 10^8^	4.2 × 10^7^	10.0
	#3	3.2 × 10^8^	7.5 × 10^6^	42.1
	average	5.8 × 10^8^	3.5 × 10^7^	23.3

For A/PR/8/34 strain, maximum TCID_50_ titers were obtained at ~30 h post infection (hpi) with both MDCK cells; for A/Uruguay/716/2007 strain, maximum TCID_50_ titers were obtained at ~30 hpi and ~43 hpi with ECACC and ATCC cells, respectively. Given the considerable inter-assay variability for TCID_50_ titers, values of three biological replicates are shown.

**Table A3:** Cell specific productivity and PP/IP ratios of influenza virus in suspension cultures with MDCK cells derived from ECACC and ATCC.

Strain	Replicate	Cell Specific Productivity					
		ECACC			ATCC		
		PP/Cell	IP/Cell	PP/IP Ratio	PP/Cell	IP/Cell	PP/IP Ratio
A/PR/8/34	#1	4376	667	7	2254	736	3
	#2	4620	253	16	5890	859	7
	#3	6640	3282	2	8404	2204	4
	#4	5809	1543	4	-	-	-
	#5	10648	2564	4	-	-	-
	average	6419	1662	7	5516	1266	5
	SD	2536	1268	6	3092	814	2
	SD%	39.5%	76.3%	84.1%	56.1%	64.3%	44.6%
A/Uruguay/716/2007	#1	602	512	1	776	22	34
	#2	2320	182	13	661	16	40
	#3	3029	117	26	738	3	261
	average	1984	270	13	725	14	112
	SD	1248	212	13	59	10	129
	SD_%_	62.9%	78.3%	93.8%	8.1%	71.1%	115.8%

PP/cell and IP/cell values were calculated when maximum TCID_50_ titers were observed. For A/PR/8/34 strain, maximum TCID_50_ titers were obtained at ~30 hpi for both MDCK cells; for A/Uruguay/716/2007 strain, maximum TCID_50_ titers were obtained at ~30 hpi and ~43 hpi with ECACC and ATCC cells, respectively. Given the considerable inter-assay variability, all values of the different biological replicates are shown. PP: physical particles (HA titers); IP: infectious particles (TCID_50_ titers). SD: standard deviation.

## References

[1] Del Rio D., Beucher B., Lavigne M., Wehbi A., Dopeso-Reyes I.G., Saggio I., Kremer E.J. (2019). CAV-2 Vector Development and Gene Transfer in the Central and Peripheral Nervous Systems. Front. Mol. Neurosci..

[2] Junyent F., Kremer E.J. (2015). CAV-2—Why a canine virus is a neurobiologist’s best friend. Curr. Opin. Pharmacol..

[3] Bru T., Salinas S., Kremer E.J. (2010). An Update on Canine Adenovirus Type 2 and Its Vectors. Viruses.

[4] Kremer E.J., Boutin S., Chillon M., Danos O. (2000). Canine Adenovirus Vectors: An Alternative for Adenovirus-Mediated Gene Transfer. J. Virol..

[5] Soudais C., Skander N., Kremer E.J. (2004). Long-term in vivo transduction of neurons throughout the rat CNS using novel helper-dependent CAV-2 vectors. FASEB J..

[6] Kochanek S. (1999). High-Capacity Adenoviral Vectors for Gene Transfer and Somatic Gene Therapy. Hum. Gene Ther..

[7] Fernandes P., Santiago V.M., Rodrigues A.F., Tomás H., Kremer E.J., Alves P.M., Coroadinha A.S. (2013). Impact of E1 and Cre on Adenovirus Vector Amplification: Developing MDCK CAV-2-E1 and E1-Cre Transcomplementing Cell Lines. PLoS ONE.

[8] Fernandes P., Peixoto C., Santiago V.M., Kremer E.J., Coroadinha A.S., Alves P.M. (2013). Bioprocess developme.nt for canine adenovirus type 2 vectors. Gene Ther..

[9] Castro R., Fernandes P., Laske T., Sousa M.F., Genzel Y., Scharfenberg K., Alves P.M., Coroadinha A.S. (2015). Production of canine adenovirus type 2 in serum-free suspension cultures of MDCK cells. Appl. Microbiol. Biotechnol..

[10] World Health Organization (1995). Cell culture as a substrate for the production of influenza vaccines: Memorandum from a WHO meeting. Bull. World Health Organ..

[11] Manini I., Domnich A., Amicizia D., Rossi S., Pozzi T., Gasparini R., Panatto D., Montomoli E. (2015). Flucelvax (Optaflu) for seasonal influenza. Expert Rev. Vaccines.

[12] Kim H., Kim Y.-H., Lee B.-Y., Lee K.S., Lee S.-J., Park M., Park Y.W. (2013). Mdck-Derived Cell Lines Adapted to Serum-Free Culture and Suspension Culture and Method for Preparing Vaccine Virus Using the Cells. Patent.

[13] Gaush C.R., Hard W.L., Smith T.F. (1966). Characterization of an Established Line of Canine Kidney Cells (MDCK). Exp. Boil. Med..

[14] Madin S.H., Darby N.B. (1958). Established Kidney Cell Lines of Normal Adult Bovine and Ovine Origin. Exp. Boil. Med..

[15] Omeir R.L., Teferedegne B., Foseh G.S., Beren J.J., Snoy P.J., Brinster L.R., Cook J.L., Peden K., Lewis A.M. (2011). Heterogeneity of the Tumorigenic Phenotype Expressed by Madin–Darby Canine Kidney Cells. Comp. Med..

[16] Dukes J.D., Whitley P., Chalmers A.D. (2011). The MDCK variety pack: Choosing the right strain. BMC Cell Boil..

[17] Lugovtsev V.Y., Melnyk D., Weir J.P. (2013). Heterogeneity of the MDCK Cell Line and Its Applicability for Influenza Virus Research. PLoS ONE.

[18] Van Wielink R., Kant-Eenbergen H., Harmsen M., Martens D., Wijffels R., Coco-Martin J. (2011). Adaptation of a Madin–Darby canine kidney cell line to suspension growth in serum-free media and comparison of its ability to produce avian influenza virus to Vero and BHK21 cell lines. J. Virol. Methods.

[19] Lohr V., Genzel Y., Behrendt I., Scharfenberg K., Reichl U. (2010). A new MDCK suspension line cultivated in a fully defined medium in stirred-tank and wave bioreactor. Vaccine.

[20] Nadeau I., Kamen A.A. (2003). Production of adenovirus vector for gene therapy. Biotechnol. Adv..

[21] Jolliffe I.T., Cadima J. (2016). Principal component analysis: A review and recent developments. Philos. Trans. R. Soc. A Math. Phys. Eng. Sci..

[22] Green D.R., Llambi F. (2015). Cell Death Signaling. Cold Spring Harb. Perspect. Biol..

[23] Harrison D.A. (2012). The Jak/STAT pathway. Cold Spring Harb. Perspect. Biol..

[24] Kawasaki T., Kawai T. (2014). Toll-Like Receptor Signaling Pathways. Front. Immunol..

[25] Lester S.N., Li K. (2013). Toll-like receptors in antiviral innate immunity. J. Mol. Boil..

[26] Cros J., Cagnard N., Woollard K., Patey N., Zhang S.-Y., Senechal B., Puel A., Biswas S.K., Moshous D., Picard C. (2010). Human CD14dim Monocytes Patrol and Sense Nucleic Acids and Viruses via TLR7 and TLR8 Receptors. Immunity.

[27] Mitchell S., Vargas J., Hoffmann A. (2016). Signaling via the NFkappaB system. Wiley Interdiscip. Rev. Syst. Biol. Med..

[28] Hughes P., Marshall D., Reid Y., Parkes H., Gelber C. (2007). The costs of using unauthenticated, over-passaged cell lines: How much more data do we need?. Biotechniques.

[29] Kluge S., Benndorf D., Genzel Y., Scharfenberg K., Rapp E., Reichl U. (2015). Monitoring changes in proteome during stepwise adaptation of a MDCK cell line from adherence to growth in suspension. Vaccine.

[30] Malm M., Saghaleyni R., Lundqvist M., Giudici M., Chotteau V., Field R., Varley P., Hatton D., Grassi L., Svensson T. (2020). Evolution from adherent to suspension systems biology of HEK293 cell line development. bioRxiv.

[31] Simeone P., Trerotola M., Franck J., Cardon T., Marchisio M., Fournier I., Salzet M., Maffia M., Vergara D., Tristan C. (2019). The multiverse nature of epithelial to mesenchymal transition. Semin. Cancer Boil..

[32] Bissinger T., Fritsch J., Mihut A., Wu Y., Liu X., Genzel Y., Tan W.-S., Reichl U. (2019). Semi-perfusion cultures of suspension MDCK cells enable high cell concentrations and efficient influenza A virus production. Vaccine.

[33] Gregersen J.-P., Schmitt H.-J., Trusheim H., Bröker M. (2011). Safety of MDCK cell culture-based influenza vaccines. Futur. Microbiol..

[34] Rodrigues A.F., Soares H.R., Guerreiro M.R., Alves P.M., Coroadinha A.S. (2015). Viral vaccines and their manufacturing cell substrates: New trends and designs in modern vaccinology. Biotechnol. J..

[35] Rodrigues A.F., Carrondo M.J., Alves P.M., Coroadinha A.S. (2014). Cellular targets for improved manufacturing of virus-based biopharmaceuticals in animal cells. Trends Biotechnol..

[36] Zhao H., Dahlö M., Isaksson A., Syvänen A.-C., Pettersson U. (2012). The transcriptome of the adenovirus infected cell. Virology.

[37] Rodrigues A.F., Formas-Oliveira A.S., Bandeira V., Alves P.M., Hu W., Coroadinha A.S. (2013). Metabolic pathways recruited in the production of a recombinant enveloped virus: Mining targets for process and cell engineering. Metab. Eng..

[38] White E. (2001). Regulation of the cell cycle and apoptosis by the oncogenes of adenovirus. Oncogene.

[39] Hendrickx R., Stichling N., Koelen J., Kuryk L., Lipiec A., Greber U.F. (2014). Innate Immunity to Adenovirus. Hum. Gene Ther..

[40] Seitz C., Frensing T., Höper D., Kochs G., Reichl U. (2010). High yields of influenza A virus in Madin-Darby canine kidney cells are promoted by an insufficient interferon-induced antiviral state. J. Gen. Virol..

[41] Le L.P., Li J., Ternovoi V.V., Siegal G.P., Curiel D.T. (2005). Fluorescently tagged canine adenovirus via modification with protein IX–enhanced green fluorescent protein. J. Gen. Virol..

[42] Fernandes P., Simão D., Guerreiro M.R., Kremer E.J., Coroadinha A.S., Alves P.M. (2014). Impact of adenovirus life cycle progression on the generation of canine helper-dependent vectors. Gene Ther..

[43] Kalbfuss B., Knöchlein A., Kröber T., Reichl U. (2008). Monitoring influenza virus content in vaccine production: Precise assays for the quantitation of hemagglutination and neuraminidase activity. Biologicals.

[44] Genzel Y., Reichl U. (2007). Vaccine production—State of the art and future needs in upstream processing. Animal Cell Biotechnology: Methods and Protocols.

[45] R Core Team The R Project for Statistical Computing. http://www.r-project.org/.

[46] Palache A.M., Brands R., Van Scharrenburg G.J.M. (1997). Immunogenicity and reactogenicity of influenza subunit vaccines produced in MDCK cells or fertilized chicken eggs. J. Infect. Dis..

[47] Gröner A.J.V. (2003). Animal Cells and Processes for the Replication of Influenza Viruses. Patent.

[48] Liu J., Shi X., Schwartz R., Kemble G. (2009). Use of MDCK cells for production of live attenuated influenza vaccine. Vaccine.

[49] Liu J., Mani S., Schwartz R., Richman L., Tabor D.E. (2010). Cloning and assessment of tumorigenicity and oncogenicity of a Madin–Darby canine kidney (MDCK) cell line for influenza vaccine production. Vaccine.

[50] Reid Y.A., Pyla Y., Cedrone E. (2010). Isolation and Characterization of MDCK Clones. ATCC Connect.

[51] Chu C., Lugovtsev V., Golding H., Betenbaugh M., Shiloach J. (2009). Conversion of MDCK cell line to suspension culture by transfecting with human siat7e gene and its application for influenza virus production. Proc. Natl. Acad. Sci. USA.

[52] Huang D., Zhao L., Tan W. (2011). Adherent and single-cell suspension culture of Madin-Darby canine kidney cells in serum-free medium. Sheng Wu Gong Cheng Xue Bao.

[53] Huang D., Peng W.-J., Ye Q., Liu X.-P., Zhao L., Fan L., Xia-Hou K., Jia H.-J., Luo J., Zhou L.-T. (2015). Serum-Free Suspension Culture of MDCK Cells for Production of Influenza H1N1 Vaccines. PLoS ONE.

